# Role of differentiated embryo-chondrocyte expressed gene 2 in immunity

**DOI:** 10.3389/fimmu.2024.1335473

**Published:** 2024-03-12

**Authors:** Yujing Li, Yinan Ma, Yang Liu, Na Tang, Wenzhu Zhang, Jingru Huo, Di Zhang

**Affiliations:** ^1^ Department of Pathology, The First Hospital of China Medical University, Shenyang, Liaoning, China; ^2^ Department of Pathology, College of Basic Medical Sciences, China Medical University, Shenyang, Liaoning, China; ^3^ Department of Pathology, Sichuan Cancer Hospital & Institute, Sichuan Cancer Center, School of Medicine, University of Electronic Science and Technology of China, Chengdu, Sichuan, China

**Keywords:** DEC2, DEC1, Bhlhe41, immunity, immune cells

## Abstract

Differentiated embryo-chondrocyte expressed gene 2 (DEC2) is a member of the basic helix-loop-helix (bHLH) subfamily of transcription factors. DEC2 is implicated in tumor immunotherapy, immune system function regulation, and autoimmune diseases. DEC2 enhances Th2 cell differentiation by regulating the IL-2 and IL-4 signaling pathways and mediates the growth of B-1a cells, thereby promoting the occurrence and development of inflammatory responses. In this study, we review the reported roles of DEC2, including the regulation of immune cell differentiation and cytokine production in various cells in humans, and discuss its potential in treating autoimmune diseases and tumors.

## 1 Introduction

DEC2, also known as BHLHE41 (class E basic helix-loop-helix protein 41)/BHLHB3 (Class B basic helix-loop-helix protein 3), is a bHLH repressor transcription factor (1), whose sequence is similar to that of BHLHE40 (class E basic helix-loop-helix protein 40)/DEC1 (Differentiated embryo-chondrocyte expressed gene 1)/STRA-13 (stimulated with retinoic acid 13). It is encoded by *DEC2*, located on the human chromosome 12p11.23-p12.1 and comprises 482 amino acid residues with a molecular weight of 50.5 kDa (2). DEC2 is classified as a transcriptional repressor based on its domain and transcriptional properties and shares a high degree of sequence homology with other bHLH transcriptional repressor subfamily members (2). The bHLH family of transcription suppressors performs diverse transcriptional repression functions involving various mechanisms (3, 4). DEC2 achieves transcriptional repression of the target gene promoter activity via DNA binding (5).

Recent studies have demonstrated the crucial role of DEC2 in regulating circadian rhythms, immune homeostasis, cell differentiation, regeneration, and metabolism (6–8). DEC2 regulates the circadian rhythm by mediating E-box-dependent transcriptional repression through various mechanisms (9). DEC2 also participates in several pathways with diverse functions that help regulate the biological behavior of immune cells and tumors. For example, DEC2 regulates the self-renewal of B-1a cells (10). In cervical cancer, DEC2 inhibits the epithelial–mesenchymal transition (EMT) and tumor metastasis through the Notch signaling pathway (11). In gastric cancer, DEC2 inhibits EMT-related metastasis by inactivating several pathways, including the ERK/NF-κB (12) and PI3K/Akt pathways (13). In addition, DEC2 regulates the differentiation of Th2 cells (7) and the self-renewal of alveolar macrophages (AMs) (14). DEC2 is also closely associated with tumors, with its transcription level serving as a marker of cancer progression (12, 13, 15–18).

Despite the complex structure and diverse immune functions of DEC2, its role in the immune system remains inadequately understood. Therefore, additional investigations are necessary to ascertain DEC2’s potential in diagnosing and treating autoimmune diseases, as well as identifying novel targets for tumor treatment. Exploring the role and potential of DEC2 in diagnosing and treating autoimmune diseases offers avenues for identifying new therapeutic directions in oncology.

## 2 DEC2 and immune cells

### 2.1 DEC2 and T cells

Naive CD4+ T cells possess the ability to undergo differentiation into distinct Th cell subsets, including Th1, Th2, and Th17 cells, based on their cytokine expression profiles and lineage-specific transcription factors (12, 13). Specifically, Th2 cells are characterized by the expression of transcription factors such as GATA-3 and type 2 cytokines, including IL-4 (19). During immune responses, the Th2 cell population can significantly expand, serving as a crucial, albeit not exclusive, source of IL-4 (15).The expression of DEC2 is mediated by the IL-4/Stat6 pathway and can be further enhanced by ICOS or IL-25 (20). DEC2 expression is necessary to maintain the continuous differentiation of Th2 cells in later stages (7). Dec2-deficient mice exhibit significant defects in Th2 immune response, and T cell-specific DEC2 transgenic mice are more prone to developing allergic airway inflammation (21). DEC2 deficiency has notable consequences on Th2 cells, leading to a substantial reduction in type 2 cytokine levels. This deficiency also results in diminished expression of key transcription factors, including GATA-3 and JunB (7).. Consequently, DEC2 is preferentially expressed in Th2 cells and plays a crucial role in their differentiation.

**Figure 1:**
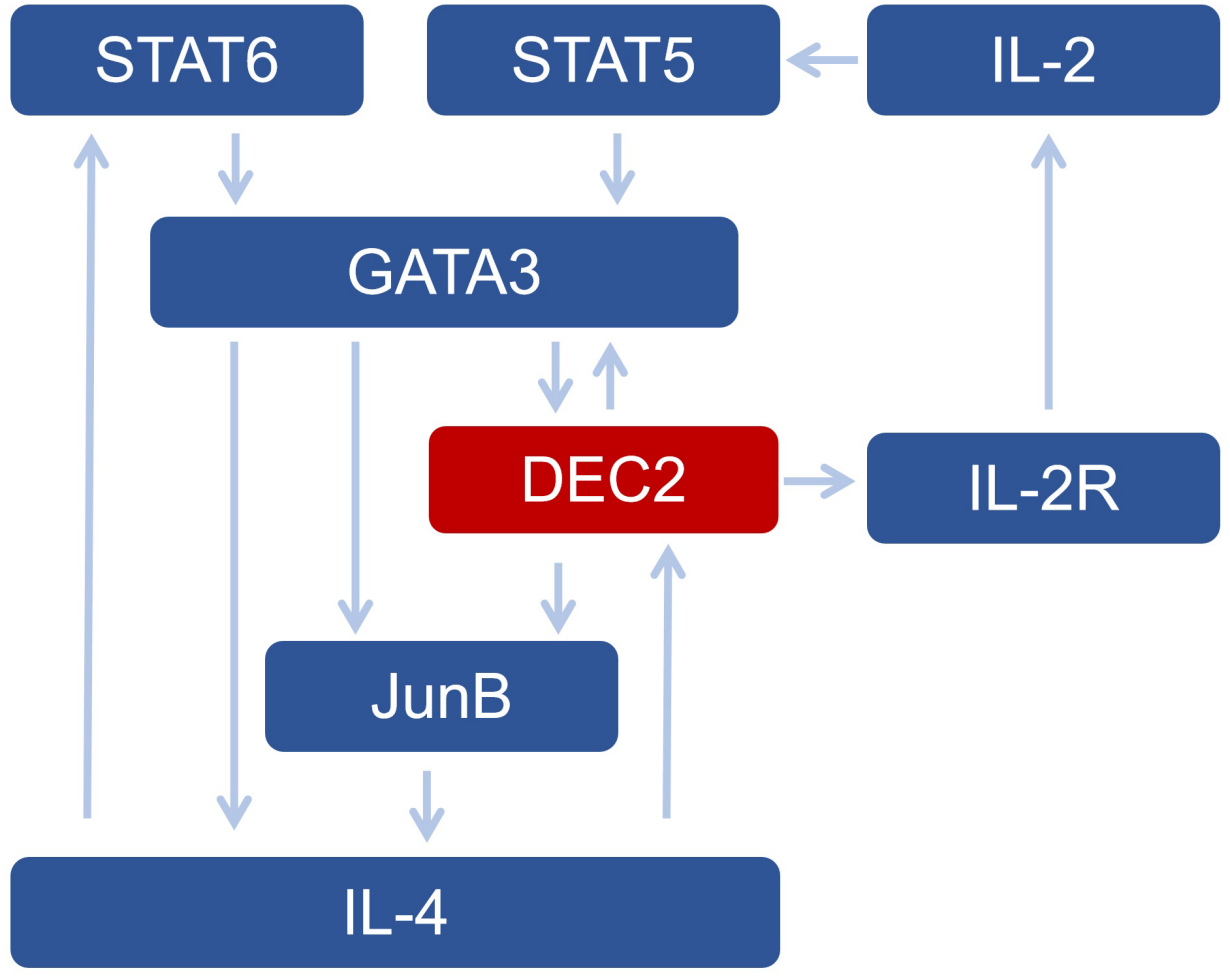
The network of DEC2 and other transcription factors in Th2 cells.

There is an autoregulatory cycle between JunB, GATA-3, and DEC2 (21). DEC2 can positively regulate GATA-3 and JunB; that is, the deficiency in type 2 cytokine expression can be alleviated by JunB or GATA-3 expression (21). In addition, DEC2 enhances the sensitivity of Th2 cells to IL-2 by enhancing CD25 expression in a STAT6-dependent manner (7). Therefore, DEC2 promotes Th2 differentiation by activating JunB and GATA-3 expression and promoting early IL-4 production. GATA-3 further regulates DEC2 expression, forming a cycle involving JunB, GATA-3, and DEC2 (21) (**Figure 1**).

IL-2 promotes IL-4 expression and T helper (Th) 2 cell differentiation via various mechanisms (19). IL-2, a 15.5 kDa cytokine secreted by antigen-activated T cells, plays a vital role in lymphocyte activation and differentiation (22). It is an essential growth factor for T cells and is associated with the initiation of immune responses (23). For example, IL-2 enhances the cytolytic activity of NK cells and tumor-infiltrating lymphocytes, promotes immunoglobulin production in activated B cells, and maintains the homeostatic proliferation of regulatory T cells (Tregs) (22). In addition, IL-2 acts on the innate lymphoid cells, regulates the differentiation of effector T cells, and affects memory T cells, effector T cells, and monocytes (22). DEC2 enhances the IL-2R-mediated signaling pathway and promotes Th2 differentiation (7).

**Figure 2:**
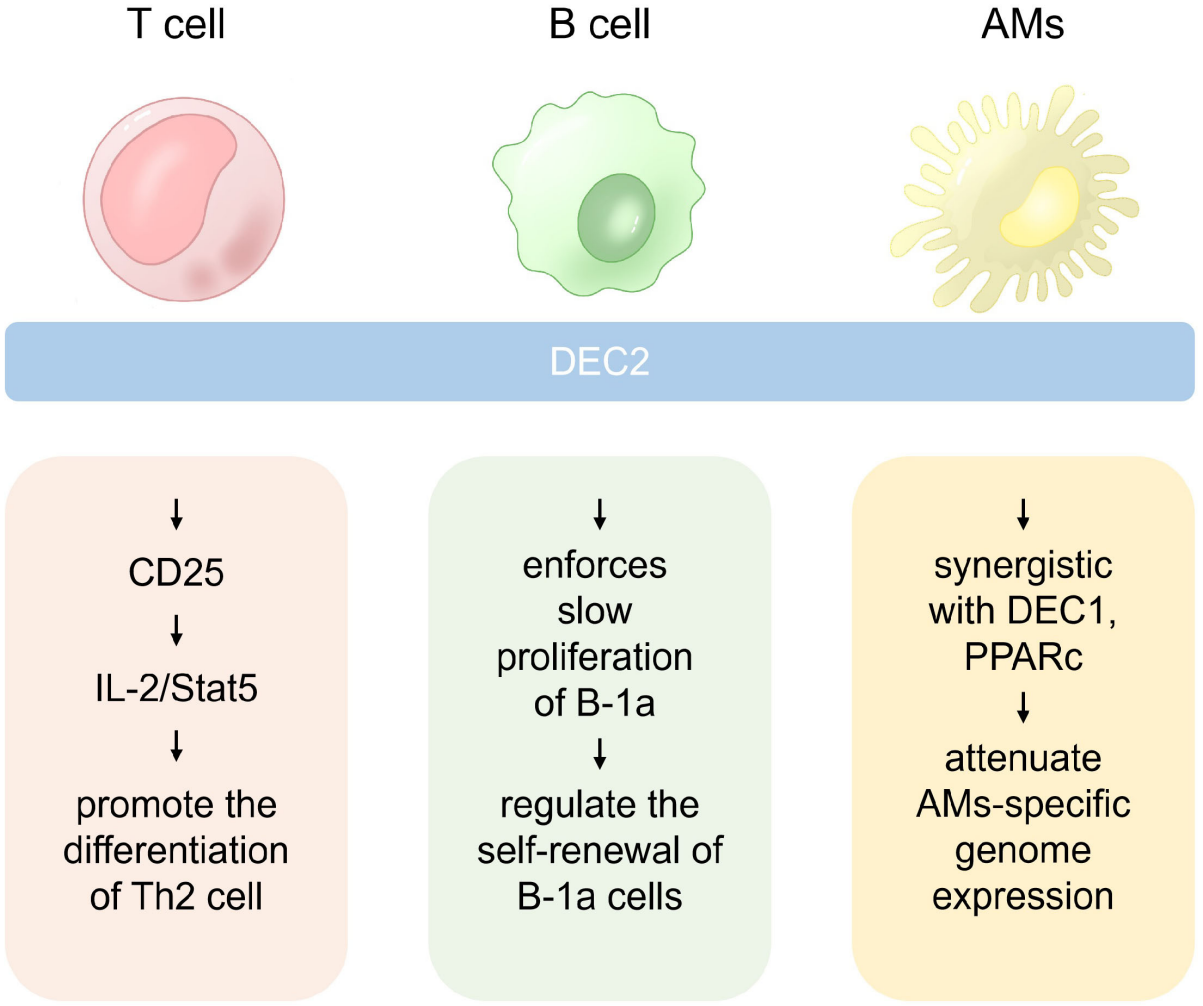
The role of DEC2 in different immune cells. In Th2 cells, DEC2 is pivotal for differentiation, while in B-1a cells, it governs self-renewal by enhancing responsiveness to pro-survival cytokines. Additionally, DEC2 is a critical regulator of alveolar macrophage (AM) self-renewal.

DEC2 induced by IL-4 signaling may help maintain high levels of IL-2R, thus facilitating Th2 cell activation at later stages (7). Therefore, DEC2 is critical in regulating the IL-2 signaling pathway and exerts a synergistic effect with IL-4 to promote Th2 cell differentiation (**Figure 2**).

Blockers of the above pathway, such as Rademikibart (24, 25), AK120 (26) and other IL-4Rα antagonists that bind to IL-4Rα, or Black Ginseng Extract (27) can effectively block the IL-4/Stat6 pathway and are indicated for the treatment of Th2-associated inflammatory diseases.

### 2.2 B cells

The B-1 cell population is an innate B lymphocyte subset first identified over 30 years ago (28). These cells, which are found in the peritoneal and pleural spaces, omentum, and spleen, are considered the first line of defense against pathogens and are involved in maintaining homeostasis in the internal environment (29). B-1 cells protect from infection, regulate Tregs during the initiation of pathogen-specific immunity, and participate in autoimmune diseases (30).

B cells can be categorized as B-1 or B-2 based on their characteristic surface molecular phenotype, and the main subsets of B-1 cells are divided into CD5^+^ B-1a cells and CD5^−^ B-1b cells (31). DEC2 is crucial in regulating B-1a cell development, self-renewal, and B cell receptor (BCR) repertoire (10, 32). The regulation of DEC2 in B1-a cells can be categorized into organ, cellular, and molecular levels. DEC2 is highly expressed in B-1a and B-1b lymphocytes, lung macrophages, and microglia (33). During B-cell development, DEC2 is expressed at low levels in pre-B, immature B, and plasma cells of the bone marrow and transitional B cells of the spleen (10). DEC2 exhibits elevated expression levels in immature B cells found in fetal and neonatal livers when contrasted with its expression in adult bone marrow. This heightened expression is correlated with an increased tendency for precursor cell production in fetuses and neonates (10). DEC2 is highly expressed in postnatal and adult B-1 cells and is upregulated during cell development.

B-1a cell numbers were significantly reduced in DEC2^−/−^ and DEC1^−/−^ DEC2^−/−^ double-knockout (DKO) mice, highlighting the critical role of DEC2 in B-1a cell differentiation and stabilization (10). Mutated B-1a cells develop abnormal cell phenotypes, accompanied by a notable alteration in the BCR repertoire. This is illustrated by the absence of the PTC-specific VH12/Vκ4 BCR (10). At the molecular level, DEC2 coordinates with DEC1 to mediate cell cycle arrest by directly inhibiting cell cycle regulators, including E2F transcription factors (10). Additionally, DEC2 also controls the self-renewal of B-1a cells by rendering B-1a cells receptive to pro-survival cytokine signaling (10, 21).

DEC2 also limits the number of B-1a cells in human adulthood (10, 34). Thus, DEC2 controls B-1a cells in multiple ways by regulating their development, BCR repertoire, and self-renewal (**Figure 2**).

### 2.3 Other immune cells

Macrophages are the first line of defense against pathogens (35). Most alveolar macrophages (AMs) differentiate after exposure to granulocyte-macrophage colony-stimulating factor (GM-CSF) produced by alveolar epithelial cells (36). When cells are exposed to GM-CSF and TGFβ, it leads to the activation of PPARc expression. This activation of PPARc expression, in turn, initiates the transcription of RNA essential for the function of AMs. The intricate process involves collaboration with other transcription factors, such as BACH1, BACH2, and CEBPb (37).

DEC2 is a regulator of AMs that perform homeostatic functions in the alveoli (14). DEC2 and DEC1 attenuate the expression of certain genes described below. Genes that comprise an “AM signature”, such as Epcam and Acaa1b, were upregulated in DEC2/DEC1-deficient AMs (14). However, the macrophage population with “a non-AM signature” showed the upregulation of widely expressed genes, including genes encoding the complement component C1q (*C1qa*, *C1qb*, and *C1qc*), transcription factor MafB (*Mafb*), cholesterol carrier apolipoprotein E (*ApoE*), and chemokine-like receptor 1 (*Cnklr1*) (14). Therefore, DEC2 emerges as a crucial regulator in AM self-renewal, serving as the guardian of characteristic genes. Its involvement in the control of tissue-specific functions of macrophages suggests likely interactions with other transcription factors (**Figure 2**).

Macrophages serve as crucial innate immune defenders within tissues, particularly in combating lipopolysaccharide (LPS)-induced periodontal inflammation (38). Activation of macrophages triggers the release of proinflammatory cytokines, including interleukin (IL)-1β. LPS can activate caspase-11 and stimulate the secretion of IL-1β, which subsequently triggers pyroptosis. He et al. (39) reported that DEC2 overexpression reduced IL-1β expression in *Porphyromonas gingivalis* LPS-induced macrophages. Consequently, DEC2 deficiency in periodontal macrophages aggravated *P. gingivalis* LPS-induced periodontal inflammation and pyroptosis.

## 3 DEC2 in immunoregulation and tumor therapy

In the tumor microenvironment, cancer cells evade host immunity through several pathways. One of the most critical components of this pathway is the immunosuppressive co-signal (immune checkpoint) mediated by programmed death receptor 1 (PD-1) and its ligand, PD-L1 (40, 41). PD-L1 binds to the PD-1 receptor expressed on T cells and tumor-associated macrophages (TAM) and blocks antitumor activity by inducing their apoptosis (42, 43). Notably, therapeutic interventions targeting these immune checkpoints, specifically PD-1 and PD-L1, have received regulatory approval for the treatment of specific malignancies (44, 45).

In this immunologically dynamic context, Tsuruta et al. reported (46) that DEC2 exhibits diurnal fluctuations in its expression within tumor-associated macrophages (TAMs), exerting periodic suppression on NF-κB-induced transactivation of the Pdcd1 gene in RAW264.7 cells. This cyclic inhibition may contribute to the diurnal expression pattern of PD-1 in TAMs. Li et al. (12) demonstrated that DEC2 could also inhibit tumor proliferation and metastasis by regulating the NF-κB pathway in gastric cancer.

## 4 DEC2 and autoimmune diseases

Autoimmune diseases, such as systemic lupus erythematosus (SLE) and rheumatoid arthritis (RA), typically involve multiple tissues and organs (47, 48). DEC2 plays a crucial role in the pathogenesis and treatment of autoimmune diseases.

RA is a common chronic inflammatory joint disease in which IL-1β is a critical pathogenic factor (49). DEC2 is closely associated with RA etiology. DEC2 increases IL-1β expression in Th2 cells and is abundantly expressed in the RA synovium (50). DEC2 directly regulates IL-1β expression in HEK293 cells and primary human fibroblasts (50). The mRNA and protein expression of DEC2 is increased in synovial fibroblasts in an NF-κB -dependent manner under the influence of TNF-α (50).

SLE is a chronic inflammatory autoimmune disease characterized by the production of large amounts of heterogeneous autoantibodies against self-antigens (51); its most dangerous clinical manifestation is lupus nephritis. Imaizumi et al. reported the possibility of a DEC2-mediated IFN-β/RIG-I/CCL5 negative feedback loop (52), which they hypothesize that may play a role in controlling renal inflammation and antiviral immune responses, leading to inflammatory kidney diseases, such as lupus nephritis. However, it is unclear whether the negative feedback loop has a pro-inflammatory or anti-inflammatory function in the pathogenesis of inflammatory kidney disease and whether it is beneficial or harmful to the host. Qi et al. (53) confirmed that miR-16 reduces the possibility of renal tissue dysplasia and glomerular injury in *Fcgr2b* ^−/−^ mice by downregulating DEC2 and inhibiting thylakoid cell proliferation.

## 5 Perspectives and conclusion

DEC2 plays vital roles in circadian and non-circadian regulation, such as cell differentiation, regeneration, and maintenance of immune homeostasis. DEC2 is involved in regulating various immune cells. In Th2 cells, DEC2 promotes production through the IL-4/Stat6 pathway. It also enhances the IL-2R-mediated signaling pathway and promotes Th2 differentiation in a Stat6-dependent manner. DEC2 promotes development, self-renewal, and BCR repertoire formation in B-1a cells at various levels. The expression of DEC2 is markedly elevated in certain lymphocytes, lung macrophages, microglia, as well as immature B cells in the fetal and neonatal liver, whereas it is comparatively lower in pre-B cells, immature B cells, adult bone marrow plasma cells, and spleen migratory B cells. DEC2 exhibits the capability to inhibit the periodicity of B-1a cell cycle, regulate their self-renewal, and maintain the balance between B-1 and B-2 cell. DEC2 can also participate in the regulation of tissue-specific functions of AMs through interaction with other transcription factors. In periodontal tissue macrophages, DEC2 deficiency exacerbates periodontal inflammation and pyroptosis. In tumor immunity, DEC2 inhibits tumor growth and migration by regulating cell cycle proteins, epithelial–mesenchymal transition, and hypoxia-inducible factors (12, 15, 18, 54, 55). In autoimmune diseases, such as RA and SLE, DEC2 expression is abnormal and promotes the production of pathogenic factors such as IL-1β. Research on DEC2 has provided novel insights into the treatment of tumors and autoimmune diseases. However, owing to the complex structure of DEC2 and the large number of regulatory factors and signaling pathways involved, most current studies on its function are at the *in vitro* level. Further investigation is required to elucidate its mechanisms of immune regulation *in vivo*.

## Author contributions

YJL: Writing – original draft. YM: Writing – original draft. WZ: Writing – original draft. YL: Writing – original draft. JH: Writing – original draft. NT: Writing – original draft. DZ: Writing – review & editing.
